# Evaluation of Water Ammonium Ion Test Kit and Its Feasibility for the Analysis of Protein in Food

**DOI:** 10.3390/molecules27154689

**Published:** 2022-07-22

**Authors:** Dan Wu, Qile Xia, Jianfeng Zhou, Xingqian Ye

**Affiliations:** 1 Department of Food Nutrition, College of Biosystems and Food Science, Zhejiang University, Hangzhou 310058, China; psu@zju.edu.cn; 2 Key Laboratory of Post-Harvest Handling of Fruits, Food Science Institute, Zhejiang Academy of Agricultural Sciences, Hangzhou 310021, China; cookxql@163.com; 3 Cixi Environmental Protection Monitoring Station, Ningbo 315300, China; zhjf_mickey@126.com

**Keywords:** ammonium ion test kit, protein, rapid determination

## Abstract

The traditional method for the determination of protein in food needs the operations of digestion, distillation, absorption, and titration; therefore, it is complicated and time-consuming and requires professional personnel. Is there a more convenient and faster detection method that can directly determine the ammonium ions in protein digestion solution to obtain the protein content of food and avoid the distillation–absorption–titration process? The feasibility of water ammonium ion test kits for food protein rapid detection was discussed here. After digestion, the protein in food transforms into ammonium ions in the digestion solution. Because of the variety of food, there are many different inorganic ions left in the food digestion solution, and at the same time, digestion agents are added in the digestion process and become potential interference factors in ammonium determination. Therefore, the detection accuracy of ammonium test kits needs to be evaluated first, including their anti-interference ability. The standard curve of ammonium was established by the test kit. When the ammonium concentration was 0.00–2.50 mg/L, the absorbance at 620 nm was linearly related to the ammonium concentration, the determination coefficient R^2^ was 0.9995, and the detection limit of this method was 0.01 mg/L. The influences of temperature, pH value, and reaction time on the test kit method were discussed. The precision was 0.90–3.33%; the repeatability was 1.71–4.86%; and the recovery rate of tap water, river water, and sea water was controlled within 90–103%. The anti-interference ability of the evaluated test kit was better than that of the national standard detection method. The test kit, combined with sample pretreatment and protein conversion formula, was used to detect protein in different types of food (milk powder, rice flour, wheat flour, soy, banana, milk, fish food, chicken food, and dog food). The results showed that there were no significant differences (ρ > 0.05) between the national method and the test kit method. The ammonium ion test kit method shortened the determination time and had higher sensitivity, showing its potential for the rapid determination of food protein.

## 1. Introduction

In traditional determination methods, protein in food is determined by transforming relevant ingredients in raw materials into ammonium ions (NH_4_^+^), which react with sodium hydroxide to generate ammonia. Ammonia is absorbed by an absorption solution through distillation and then neutralized by acid–base titration. The concentration of the target determination substance is indirectly obtained by calculating ammonium ions. The distillation–absorption–titration operation requires professional and technical personnel, and the process requires a professional distillation device. The operation is tedious, and the determination time is long, often taking 1–2 h. This method is not suitable for rapid determination. There are many detection methods of ammonium ions. Is there a more convenient and faster detection method that can directly determine the ammonium ions in protein digestion solution to obtain the protein content of food? This topic is very worthy of study and discussion.

Zhu and Dong et al. reviewed the determination methods of ammonium ions [1,2]. The current methods of ammonium ion determination at home and abroad include spectroscopy, chromatography, electrochemistry, titration, surface plasmon resonance, the enzymatic method, etc. Among these methods, the spectral methods include spectrophotometry (the Nessler’s reagent method, the indophenol blue method (including the salicylic acid method), the subbromate method), fluorescence spectrometry, the chemiluminescence method, and so on. The chromatographic methods include ion chromatography, gas chromatography–mass spectrometry, solid phase extraction–high performance liquid chromatography, and so on. The electrochemical analyses include the electrochemical sensor method, the conductance method, the current method, the voltammetry method, and so on. Among these methods, fluorescence spectroscopy, chemiluminescence, surface plasmon resonance, and chromatography can be used only for laboratory analysis because of their need for expensive and large instruments. They are not suitable for rapid determination of ammonium ions. The titration method requires water predistillation, which is complicated and time-consuming and requires professional personnel. The spectrophotometric methods of the Nessler’s reagent method and the salicylic acid method have potential in the rapid determination of NH_4_^+^, as the detection reagents of these methods are easy to obtain and carry. The electrode electrochemical method is also suitable for rapid determination, but it is easily disturbed by ions present in water and its determination limit is high, so its application is limited.

**Figure 1:**
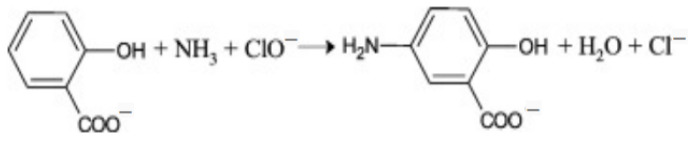
Reaction principle of salicylate method.

Zhou et al. [3] compared four determination methods of total ammonium ions, namely the Nessler method, the phenate method, the salicylate method, and the ammonia electrode method, and confirmed that salicylate spectrophotometry had the best accuracy and precision among the four methods. The salicylate method is also one of the fastest rapid determination methods for analysis of ammonium ions developed in recent years. The principle of the salicylate method is as follows:

In the presence of sodium nitroferricyanide, ammonium reacts with hypochlorite to produce monochloramine at first. Then, the monochloramine reacts with salicylate to form blue-green-colored 5-aminosalicylate, which is determined by colorimetry at 600–700 nm (Figure 1). The method has the characteristics of sensitivity, stability, environmental protection, and low determination limit. Its color development process and anti-interference capability for calcium ions (Ca^2+^) and magnesium ions (Mg^2+^) can be optimized by adjusting the amounts of salicylate and hypochlorite and adding catalysts and masking agents [4,5].

The salicylate method in the field of rapid determination of ammonium ions is mainly reflected by its combined use with a flow analyzer. Its determination time has ranged from 1.58 to 10 min per sample, and determination objects have included river water, rainwater, seawater, tap water, well water, mountain spring water, and so on [6,7,8,9,10]. A small number of salicylate test paper methods have also been reported [11].

Ammonium ion test kits are test kits used to determine the concentration of ammonium ions in a sample solution. At present, kit products on the market use two main colorimetric methods for the determination of ammonium, namely the Nesslerization and indophenol blue (IPB) methods. Nessler’s reagent consists of mercury(II) iodide, which is harmful to the environment, and the method is always interfered with by Ca^2+^ and Mg^2+^ [1]. Table 1 compares some of the commercially available IPB test kits available for selection, with detection times ranging from 5 to 30 min.

Can the concentration of ammonium in digestion solution be directly measured, without distillation, with an ammonium ion test kit after digestion of food? In this paper, an ammonium ion test kit based on the salicylate method was used for rapid determination of protein in food. The feasibility of ammonium ion test kits for protein detection techniques is discussed. First, the standard curve of ammonium ion determination in water was established. The influencing factors of this method were discussed, which included temperature, pH value, and reaction time. The determination characteristics of the ammonium ion test kit were evaluated for the aspects of precision, repeatability, recovery, and anti-interference ability. According to the established standard curve, combined with the sample pretreatment method and protein conversion formula, the protein determination of food with this test kit was evaluated.

## 2. Results and Discussion

### 2.1. The Ammonium Standard Curve for the Test Kit Method

**Figure 2:**
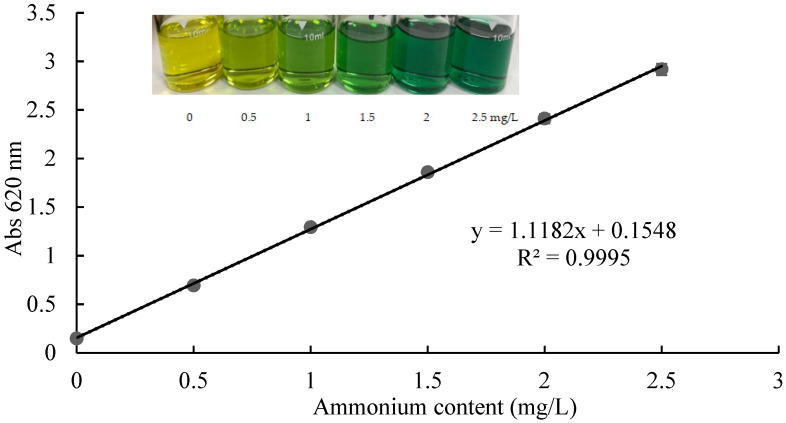
The ammonium standard curve and color change of the reaction for the test kit.

The standard curve of ammonium solution was obtained after 15 min of reaction at room temperature (Figure 2). When the ammonium concentration was 0.00–2.50 mg/L, the absorbance at 620 nm was linearly related to the ammonium concentration. The standard curve equation was as follows:

y = 1.1182x + 0.1548 (1)

where x is ammonium content and y is the absorbance at 620 nm. The determination coefficient R^2^ was 0.9995. The linear regression was good. According to the technical guidelines for the development of environmental monitoring analytical method standards [12], the detection limit of spectrophotometry can be calculated by the following formula:

*MDL* = 0.01/*b* (2)

where *MDL* means the method detection limit and b is the slope of regression line. The slope of the standard curve of the test kit method was 1.1182, so its *MDL* was as follows:

(3) $$MDL=\frac{0.01}{1.1182}=0.01$$

Therefore, the detection limit of this method was 0.01 mg/L. This corresponded to the minimum detection limit of the solid reagent method reported by Wu [13]. The salicylate method is one of the indophenol blue (IPB) methods, which are based on the Berthelot reaction first reported in 1859 [1]. Many modifications have been performed, including to the reactants, the catalysts, the masking agents, and so on [1,14,15,16,17]. The final colors of the reaction products are blue, blue-green, or green, depending on the different reagents and reaction conditions. In this method, the final reaction solution changed from yellow to green (Figure 2). According to the Beer–Lambert law [18], there is a relationship between the absorbance of electromagnetic radiation and analyte concentration. By mathematical modeling, the concentration of reactants can be derived from measured absorbance values. As shown by Equation (1) and the determination coefficient, the test kit method conformed to the Beer–Lambert law, which provides a basic theory for the analysis of ammonium.

### 2.2. The Influences of Time, Temperature, and pH on the Test Kit Method

**Figure 3:**
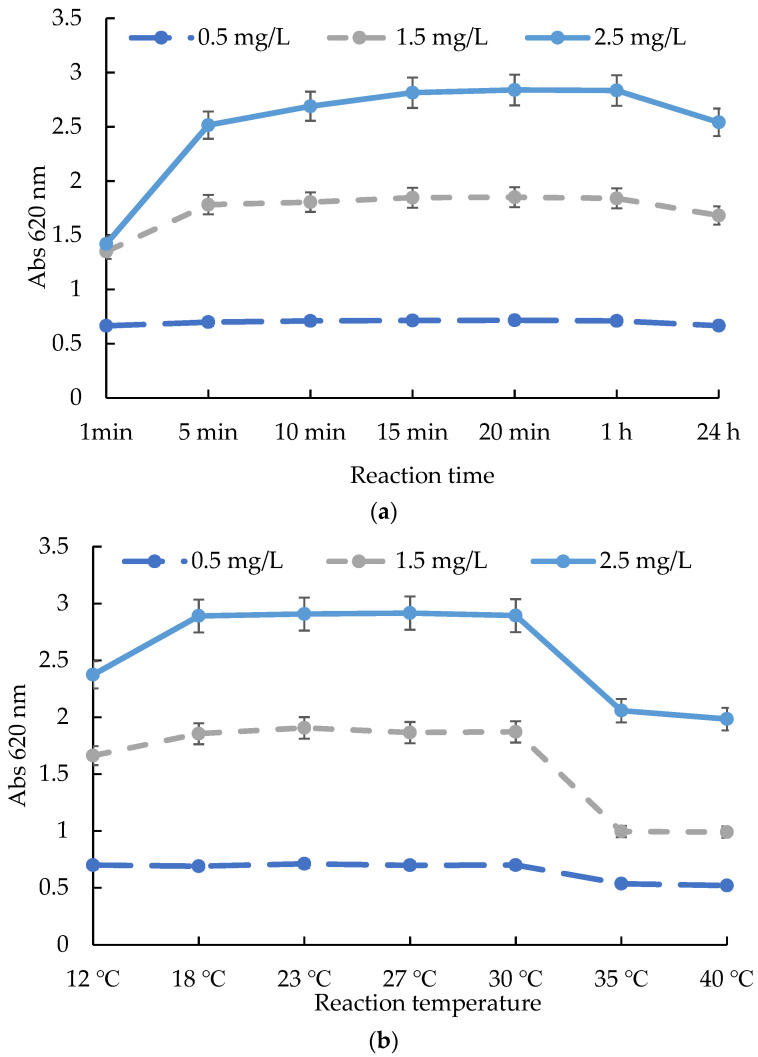

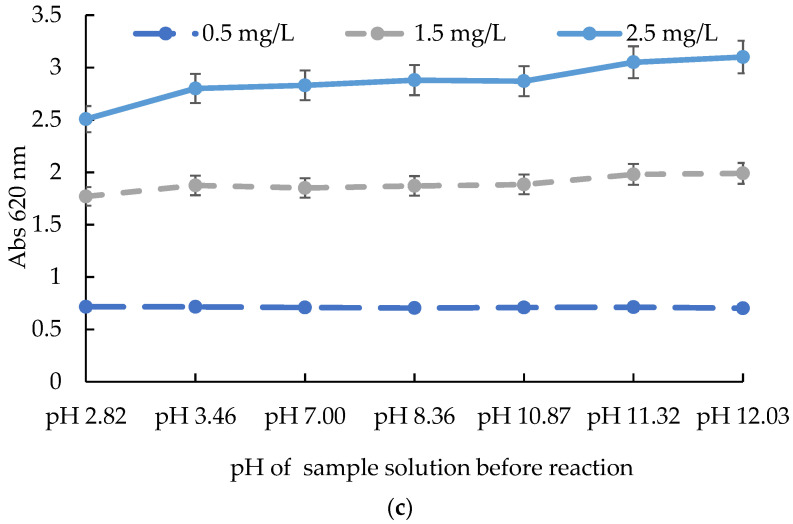
The influences of time, temperature, and pH on the test kit method: (**a**) time; (**b**) temperature; (**c**) pH.

Ammonium solutions with concentrations of 0.5, 1.5, and 2.5 mg/L were selected to study the influences on the reaction of time, temperature, and pH value. Figure 3a shows the variation in the characteristics of the reaction solution at 1 min, 5 min, 10 min, 15 min, 20 min, 1 h, and 24 h. The results showed that reaction equilibrium times differed with different ammonium concentrations. Low-concentration solutions of 0.5 and 1.5 mg/L took 5–10 min to reach equilibrium, while the high-concentration solution of 2.5 mg/L needed 15 min. After 15 min of reaction, the absorbance value of the reaction solution was basically unchanged within 1 h, and there was no significant difference in the 95% confidence interval. When the reaction reached 24 h, the absorbance decreased. The largest drop in the absorbance value was more than 10%. Therefore, a reaction time of 15 min was appropriate. Figure 3b shows the variation in the characteristics of the reaction solution at 12 °C, 18 °C, 23 °C, 27 °C, 30 °C, 35 °C, and 40 °C. The results showed that too-low and too-high reaction temperatures had negative effects on the reaction. When the temperature was between 18 and 30 °C, the reaction was relatively stable, and the variation error of reaction was not more than 10%, especially between 23 and 27 °C, where the variation error was less than 5%. Figure 3c shows the variation in the characteristics of the reaction solution at pH 2.82–12.03. The results showed that the test kit was suitable for a wide pH range. When the sample solution’s pH was between 2.82 and 12.00, the variation error of the reaction was not more than 10%, especially between pH 3.46 and 10.87, where the variation error was less than 5%.

### 2.3. Precision and Repeatability of the Test Kit Method

The absorbance values of five groups of standard solution with different concentrations were measured and substituted into the standard curve of ammonium to calculate the concentration value. Then, the error between the real and calculated values was calculated. The precision and repeatability of the kit method were studied, and the results were shown in Table 2. The standard deviations of the three laboratories were 0.94–1.74%, 1.19–3.33%, and 0.90–2.29%. Repeatability analysis for the three laboratories showed that the standard deviations of the repeatability were between 1.71 and 4.86% and did not exceed 5%. Zhu et al. used an ammonia nitrogen kit developed by American CHEMetrics to test ammonium in water and compared the results with the national standard method. They found that the relative deviation of the stability of the kit method was 5.98%, while that of the national standard method was 1.03% [19]. Wu et al. used the solid reagent method to test ammonium in water and compared the results with the national standard method. They found that the precision of the solid reagent method was not more than 0.95%, while that of the national standard method was not more than 1.10% [13].

### 2.4. The Recovery of the Test Kit Method

In order to study the validation of the method, the recoveries of three kinds of water samples were studied. Tap water, river water, and artificial sea water [20] were taken for recovery tests. The recovery rates were calculated and are shown in Table 3. The results showed that the recovery rate of ammonium was between 91.80 and 103.00% for different water samples. Seawater with different dilutions was used to evaluate the applicability of the method for water with different salinity. The recovery of seawater with different dilutions was in the range of 90.00–99.70%. The ammonium ion test kit based on salicylate method had the potential to apply to water samples with different salinity.

Ammonium is a nutrient pollutant in water. If the concentration is too high, the water quality deteriorates, and the ecosystem is thrown out of balance [21]. The concentration of ammonium in water should be maintained below 0.20 mg/L to ensure the healthy growth of aquatic organisms; when the concentration of ammonia nitrogen exceeded 2.00 mg/L, the creatures in the water showed poisoning symptoms and even death [22]. Therefore, the detection of ammonium is very important. However, because of the different salinity of natural water samples, there is deviation in the ammonia nitrogen line. Huangpu found that when the salinity of water samples increased from 0 to 35 g/kg, the slope of the ammonium working curve increased from 1323 to 2165, about 1.64 times [23]. Calcium and magnesium plasma in water can also affect the determination of ammonium [24,25]. In [25], water sources were divided into fresh water, brackish water, and salt water. The degree of water mineralization ranged from 100 to 40,000 mg/L, and the salinity ranged from 0.2 to 10 g/kg [25]. Therefore, the standard ammonium curve based on pure water could not replace the curve based on actual water with unknown composition, there was measurement deviation. Some national standards described that if the salinity or the calcium or magnesium content or is high in water, it must be pretreated, and that if the salinity is higher than 3%, ammonium-free seawater must be used for the standard curve [26,27].

**Figure 4:**
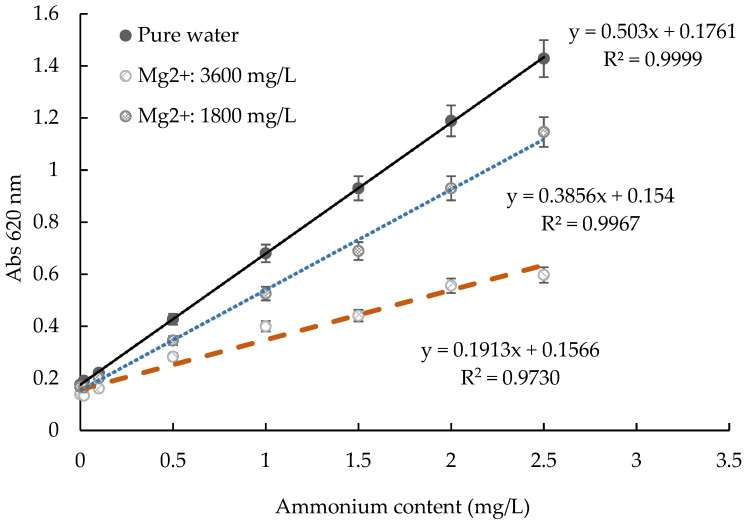
Comparison of the changes in the ammonium working curve with different Mg^2+^ concentrations for a test kit from HACH Company.

**Figure 5:**
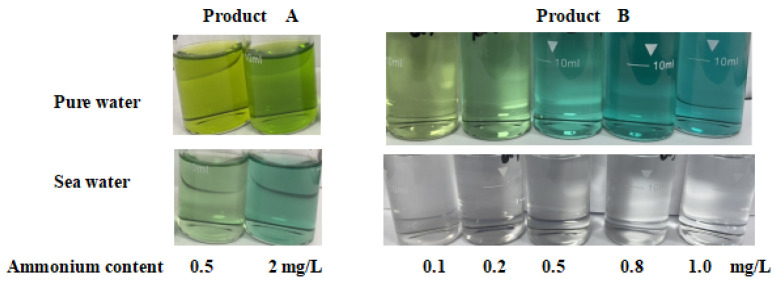
Comparison of the color changes of ammonium test kits from China (Product A: test kit suitable for low-hardness water from Zhejiang Lohand Environmental Technology Co. Ltd., Hangzhou, China; Product B: test kit from Guangdong Huankai Microbial Technology Co., Ltd., New York, NY, USA).

Several different kit products were investigated. It was found that some kits had low recovery rates for some natural water samples with high Ca^2+^ and Mg^2+^ without distillation or dilution, such as seawater (Figure 4 and Figure 5). As shown in Figure 4, as the Mg^2+^ concentration increased, the slope of the ammonium curve decreased from 0.503 to 0.191. This would lead to lower results than the real value. As shown in Figure 5, when the same amount of ammonium ions was added to pure water and ammonia-free seawater, different colors appeared, and sometimes no color appeared at all. The studies had shown that these kits had different reaction characteristics for different composition solution and needed to be further optimized to accommodate different samples.

The test kits shown in Figure 5 were mainly intended for portable and rapid detection. The field of application for test kits would be greatly expanded if their anti-interference ability were enhanced. The ammonium test kit investigated herein had such potential.

### 2.5. The Influence of Different Ions in Water for Test Kit Method

Based on 0.5 mg/L ammonium standard solution, the influence of coexisting cations and anions on ammonium determination was studied. The results are shown in Table 4. According to the coexisting ions in Appendix B of the national standard determination method HJ 536-2009 [26], if tartrate is used as a masking agent, when 8 mL of water sample contains 4 µg ammonium, the allowable coexisting ions are 500 µg Ca^2+^, 500 µg Mg^2+^, 50 µg Al^3+^, 20 µg Mn^2+^, 250 µg Cu^2+^, 50 µg Pb^2+^, 100 µg Zn^2+^, 50 µg Cd^2+^, 200 µg Cr^6+^, 100 µg Mo^6+^, 50 µg Co^2+^, 1000 µg Ni^2+^, 500 µg V^5+^, 50 µg Ag^+^, 100 µg As^3+^, 20,000 µg SO_4_^2−^, 500 µg PO_4_^3−^, 500 µg NO_3_^−^, 200 µg NO_2_^−^, 500 µg F^−^, and 100,000 µg Cl^−^. This means that if tartrate is used as a masking agent, when the ammonium content is 0.5 mg/L, the allowable coexisting ions are 62.5 mg/L Ca^2+^, 62.5 mg/L Mg^2+^, 6.25 mg/L Al^3+^, 2.5 mg/L Mn^2+^, 31.25 mg/L Cu^2+^, 6.25 mg/L Pb^2+^, 12.5 mg/L Zn^2+^, 6.25 mg/L Cd^2+^, 25 mg/L Cr^6+^, 12.5 mg/L Mo^6+^, 6.25 mg/L Co^2+^, 125 mg/L Ni^2+^, 62.5 mg/L V^5+^, 6.25 mg/L Ag^+^, 12.5 mg/L As^3+^, 2500 mg/L SO_4_^2−^, 62.5 mg/L PO_4_^3−^, 62.5 mg/L NO_3_^−^, 25 mg/L NO_2_^−^, 62.5 mg/L F^−^, and 12,500 mg/L Cl^−^. It was shown that the test kit method had certain advantages in comparison with the national standard determination method. For a sample solution in the presence of separate interfering ions of calcium, magnesium, aluminum, manganese, lead, zinc, cadmium, molybdenum, nickel, vanadium, tin, arsenic, sulfuric acid root, nitrate, nitrite, fluorine, and chlorine, this method had superior anti-interference ability to the national standard method. It had 20 times more anti-interference ability than the allowable coexistence value marked in Appendix B of the method of HJ 536-2009 in regard to 11 of the items listed above [26]. Comparing the influence of Mg^2+^ on a test kit from HACH Company (shown in Figure 4) and that used in the proposed method, the HACH test kit had about 20% measurement error at 0.5 mg/L NH_4_^+^ with 1800 mg/L Mg^2+^ coexisting, but the proposed method had no more than 10% measurement error.

In the determination of protein, the sample needs to be digested or extracted first, and sulfuric acid, copper sulfate, and potassium sulfate must be added [28,29]. At the same time, digestion decomposes only organic matter; there is much inorganic matter in digestion solution. Without predistillation, the digestion solution contains high concentrations of unknown interfering ions. These ions may interfere with the determination of ammonium in the sample.

According to the discussion of the recovery and the influence of ions in Section 2.4 and Section 2.5, this kit method had advantages in anti-interference performance compared with other products. Therefore, this kit method was applied to the determination of protein.

### 2.6. Comparison of National Method and Test Kit Method for Analysis of Food Protein

The methods of protein determination in food and feed include the Kjeldahl method, the photometric method, the combustion method, near infrared spectroscopy, and so on [18,30,31,32]. The Kjeldahl method is the most classical method with the widest application range. Spectrophotometry is the simplest and fastest determination method, but only low salinity samples are suitable, as the method is otherwise easy to disturb. Combustion is suitable for samples with a protein content of more than 10% [30,32]. After the samples were pretreated, the classical method and the ammonium ion test kit method were used to determine the related items, and the t test method was used to evaluate the homogeneity of variance of the methods. The results are shown in Table 5. The *ρ* values of the two methods were all greater than 0.05 in the determination of the protein in all of the tested samples, so there was no significant difference between the two methods.

The ammonium ion test kit method avoided the time-consuming processes of distillation and neutralization titration in the traditional method. It could synchronously determine multiple samples within 15 min, which would be beneficial to the development of rapid determination technology for food protein. At the same time, the ammonium ion detection limit of this method was 0.01 mg/L (Section 2.1). In combination with the protein conversion formula in Equation (4), without dilution, if F takes the value of 5.18 (according to GB5009.5-2016 [28]), the detection limit of protein in food can be calculated to 5 mg/100 g when the sampling amount is 1.0 g. That is, when the sampling amount is 5.0 g, the detection limit of protein is 1 mg/100 g, which sensitivity is eight times that of the Kjeldahl method in GB5009.5-2016 [28]. The ammonium ion test kit method is a potential method for rapid analysis for protein. However, the number of samples explored in this experiment was small, so further experiments and exploration are needed to apply this method to more food types.

## 3. Materials and Methods

### 3.1. Materials

Evaluation of water ammonium ion test kits: an ammonium ion test kit (ATK) based on the indophenol blue method with salicylate that was suitable for high-hardness water was purchased from Zhejiang Lohand Environmental Technology Co. Ltd. (China). Ammonium chloride, superior pure, was purchased from Sinopharm Chemical Reagent Co., Ltd. Chemicals used for the allowable coexistence of the interfering ions calcium chloride, magnesium chloride, aluminum nitrate, manganese chloride, copper sulfate, lead acetate, zinc sulfate, cadmium chloride, potassium dichromate, sodium molybdate, cobalt sulfate, nickel sulfate, sodium metavanadate, silver nitrate, sodium sulfate, sodium phosphate, sodium nitrate, sodium nitrite, sodium fluoride, and potassium chloride were all of analytical grade. They were purchased from Sinopharm Chemical Reagent Co. Ltd. Arsenic solution (analytical standard, 1000 mg/L) was purchased from Shanghai Aladdin Biochemical Technology Co.

Chemicals used for sample pretreatment of food protein: copper sulfate, potassium sulfate, sulfuric acid. Chemicals used for national methods of food protein [12,13]: boric acid solution (20 g/L and 10 g/L in water), sodium hydroxide (40 g/L and 400 g/L in water), bromocresol green solution (1 g/L in 95% ethanol), methyl red solution (1 g/L and 2 g/L in 95% ethanol), and hydrochloric acid solution (0.10 mol/L, 0.05 mol/L in water). Milk powder was purchased from Nestle Hulunbeir Ltd. Rice flour was purchased from Jiangxi Guangbaicheng Food Co., LTD. Wheat flour, soy flour, banana, milk, fish food, chicken food, and dog food were purchased from local farmers markets.

### 3.2. Ammonium Standard Curve for the ATK

Ammonium standard solution (ρ = 1000 mg/L): ammonium chloride was dried at 105 °C to constant weight, weighed at 3.8190 g, dissolved in an appropriate amount of water, transferred to a 1000 mL volumetric bottle, and diluted with water to volume.

Standard solution for ammonium (ρ = 0, 0.5, 1.0, 1.5, 2.0, 2.5 mg/L): the standard ammonium reserve solution (ρ = 1000 mg/L) was diluted to 100 mg/L, and then 0, 0.5, 1.0, 1.5, 2.0, and 2.5 mL of the solution were transferred into 100 mL volumetric flasks with a pipettor (Thermofisher Scientific Co., Ltd., Waltham, MA, USA) and diluted with water to volume.

Preparation of ammonium standard curve and sample determination: an ammonium ion test kit suitable for high-hardness water from Zhejiang Lohand Environmental Technology Co.Ltd was selected to build the ammonium standard curve. According to the operating guide, at room temperature, 6 mL ammonium test buffer and 4 mL ammonium standard solution (0, 0.5, 1, 1.5, 2, 2.5 mg/L) or sample solution were added into the colorimetric bottle. The bottle was shaken up and down 10 times, and then a pack of ammonium salicylate power was added, after which the bottle was shaken up and down 20 times. The reaction stood for 15 min, and then the absorbance at 620 nm was detected on an LH-M900 multiparameter water quality analyzer (Zhejiang Lohand Environment Technology CO,. LTD., Jiubao Town, China). The standard curves were plotted with the average data of five parallel groups.

### 3.3. Evaluation of the Influences of Time, Temperature, and pH on the ATK

The influence of reaction time: according to the ATK operating guide, at room temperature, the ammonium standard solution (0.5, 1.5, 2.5 mg/L) was selected to react with the test kit. The reaction stood for 1 min, 5 min, 10 min, 15 min, 20 min, 1 h, and 20 h, and the absorbance at 620 nm was detected for evaluation.

The influence of reaction temperature: according to the ATK operating guide, the ammonium standard solution (0.5, 1.5, 2.5 mg/L) was selected to react with the test kit at different temperatures. The reaction stood for 15 min, and the absorbance at 620 nm was detected for evaluation.

The influence of the pH of the sample solution: according to ATK operating guide, the ammonium standard solution (0.5, 1.5, 2.5 mg/L) was preadjusted to pH 2.82–12.03, and reacted with test kit at room temperature. Let the reaction stand for 15 min and detected the absorbance at 620 nm to evaluate.

### 3.4. Precision and Repeatability Evaluation of the ATK

An ammonium ion test kit suitable for high-hardness water from Zhejiang Lohand Environmental Technology Co.Ltd was evaluated here. The ammonium standard solution (0.5, 1, 1.5, 2, 2.5 mg/L) was determined 5 times with the test kit, and the standard deviations was obtained for precision. There were three laboratories involved, and repeatability was evaluated by the standard deviation of the measured data from the three laboratories.

### 3.5. Evaluation of the Recovery of and the Influence of Ions on the ATK

Recovery evaluation: three kinds of water samples (tap water, river water, and artificial sea water) were taken for recovery testing, and seawater samples with different dilutions were used to evaluate the applicability of the method to water with different salinity. These water samples, without ammonium ions added and with ammonium ions added at 0.5 mg/L, 1.0 mg/L and 2.0 mg/L, were detected by the ATK: according to the test kit’s operating guide, at room temperature, 6 mL ammonium test buffer and 4 mL interfering ion water sample were added into the colorimetric bottle, which was shaken up and down 10 times. Then, a pack of ammonium salicylate power was added, and the bottle was shaken up and down 20 times. The reaction stood for 15 min, and the absorbance at 620 nm was detected.

Ion influence test: interfering ion solutions were prepared from aqueous solutions of different chemicals reagents (see Section 3.1 for details). According to the test kit’s operating guide, at room temperature, 6 mL ammonium test buffer and 4 mL interfering ion water sample were added into the colorimetric bottle, which was shaken up and down 10 times. Then, a pack of ammonium salicylate power was added, and the bottle was shaken up and down 20 times. The reaction stood for 15 min, and the absorbance at 620 nm was detected.

### 3.6. Protein Analysis

Sample pretreatment [28,29]: 1.00 g solid sample, 0.40 g copper sulfate, 6.00 g potassium sulfate and 20.00 mL sulfuric acid in were weighed in a digestion tube and put into a digestion furnace (Shanghai Xingjia Electronic CO,. LTD., China) for digestion. When the furnace temperature reached 420 °C, digestion continued for 1 h until the reaction solution in the digestion tube was green and transparent. Then, heating was stopped, and the digestion tube was removed. After the reaction solution was cooled to room temperature, 20 mL water was added, the reaction solution was moved into a 100 mL volumetric bottle, and the sample was diluted with water to volume.

Traditional methods for the determination of protein in milk powder, rice flour, wheat flour, soy four, banana, and milk referred to GB 5009.5-2016, “National Standard for Food Safety—Determination of Protein in Food—Kjeldahl Method” [28]. Traditional methods for the determination of protein in fish food, chicken food, and dog food referred to GB/T 6432-2018, “Determination of Crude Protein in Feeds—Kjeldahl Method” [29].

Test kit method for protein determination: 10 mL supernatant of the sample solution was taken after the above pretreatment, 50 mL pure water was added, and the pH of the resulting mixture was adjusted to about 5 with sodium hydroxide. The mixture was put into a volumetric bottle and diluted with water to volume. During the determination process, the solution was also diluted according to actual needs. The concentration of ammonium ions in the final diluent was determined with reference to the method of the ammonia standard curve (3.2).

Calculation of protein content for the test kit (protein conversion formula):

(4) $$X=\frac{c*f}{10}*F$$

where

X—protein content (g/100 g);
F—protein conversion coefficient (refer to GB 5009.5-2016 [28]);
c—nitrogen content in sample solution to be measured (mg/L);
f—dilution ratio.

### 3.7. Statistical Analysis

The Data Processing System (DPS) software v13.5 was applied to fix the experimental data and establish the mathematical model [33].

## 4. Conclusions

The standard curve of ammonium ion determination by a test kit was established herein. The influences of reaction time and temperature and the pH value of the water sample on the determination were evaluated. There were no significant differences in the 95% confidence interval with reaction times between 10 and 60 min. When the reaction temperature was between 18 and 30 °C and the pH of the solution was between 2.82 and 12.03, the error of the absorbance value of the reaction solution was within 10%. The standard curve equation was y = 1.1182x + 0.1548, the determination coefficient R^2^ was 0.9995, the determination range of ammonium was 0.00–2.50 mg/L, and the detection limit was 0.01 mg/L. The standard deviations of three laboratories were 0.94–1.75%, 1.19–3.33%, and 0.90–2.29%. Repeatability analysis for these three laboratories showed that the standard deviations were between 1.71 and 4.86% and did not exceed 5%. The recovery rates of tap water, river water, and sea water were controlled within 90–103%. Samples of milk powder, rice flour, wheat flour, soy, banana, milk, fish food, chicken food, and dog food were pretreated; the sample solution were diluted to appropriate multiples; and the protein contents of the samples were determined by the investigated method using an ammonium ion test kit compared with the classic method. The results showed that there was no significant difference (ρ > 0.05) between the classical and test kit methods. Compared with the traditional distillation method, the test kit method needed no distillation device, effectively shorted the determination time from 60–120 min to 15 min, and had higher sensitivity.

This method simplified the determination procedure, shortened the experimental time, and improved efficiency. Of course, there only a few samples of food were examined herein. These cannot represent all samples, so the test kit method needs to be further verified and explored. The experimental operation of test kit method was more convenient and had lower cost and higher sensitivity than the standard method. It has potential applications in the rapid determination of protein content in food.

## Figures and Tables

**Table 1:** Some ammonium ion test kits.

Analytical Method	Manufacturer	Detection Range mg/L	Advantages and Disadvantages
IPB-salicylate	Macherey-Nagel GmbH& Co. KG (Düren, Germany)	0.05–3.00	Seawater needs 1 + 1 dilution; turbidity makes the measurements too high
		0.01–2.50	Suitable for seawater; turbidity makes the measurements too high
	Kyoritsu Chemical-Check Lab., Corp (Tokyo, Japan)	0.5–20	Apply to industrial wastewater, sea water
		0.2–10	Suitable for cleaner water, river water, groundwater, and drinking water; not suitable for wastewater
	Hach Company (Loveland, CO, United States)	0.02–2.5	Iron ions interfere with the determination; monoammonium chloride, hydrazine, amino-acetic acid, turbidity, and chromaticity may cause high measurement results
		0.4–50	
		0.01–0.50	
	Zhejiang Lohand Environmental Technology Co. Ltd. (Jiubao Town, China)	0.01–2.00	Suitable for low-hardness water
		0.02–2.50	Suitable for high-hardness water, wastewater
	Guangdong Huankai Microbial Technology Co., Ltd. ((Guangzhou, China)	0.01–1.00	Not suitable for sea water
		0.4–50	Suitable for clean seawater; detection limit is high
IPB-phenol	Macherey-Nagel GmbH& Co. KG (Germany)	0.5–8.0	Seawater needs to be diluted more than 10 times for determination
IPB-phenol	Macherey-Nagel GmbH& Co. KG (Germany)	0.1–2.5	Seawater needs to be diluted more than 10 times for determination

**Table 2:** Precision and repeatability of test kit method.

Concentration (mg/L)	Lab (A) Precision (*n* = 5)		Lab (B) Precision (*n* = 5)		Lab (C) Precision (*n* = 5)		Repeatability (*n* = 15)	
	Mean /(mg/L)	RSD %	Mean /(mg/L)	RSD %	Mean /(mg/L)	RSD %	Mean /(mg/L)	RSD %
0.5	0.48	0.94	0.48	2.28	0.50	0.90	0.49	1.71
1	1.02	1.08	0.93	2.37	0.99	1.52	0.98	3.86
1.5	1.52	1.35	1.51	3.33	1.54	1.34	1.52	2.52
2	2.02	1.15	1.91	1.19	1.97	1.01	1.96	4.86
2.5	2.47	1.74	2.43	2.07	2.53	2.29	2.48	4.71

**Table 3:** The recovery of the test kit method (*n* = 5).

Sample	Background Concentration (mg/L)	Concentration after Addition (mg/L)			Recovery (%)		
		0.5 mg/L	1 mg/L	2 mg/L	0.5 mg/L	1 mg/L	2 mg/L
Tap water	0	0.490 ± 0.010	0.950 ± 0.080	2.060 ± 0.120	97.40	95.40	103.00
River water	0.05	0.480 ± 0.010	0.990 ± 0.060	1.940 ± 0.080	96.60	98.70	97.05
Artificial seawater	0	0.459 ± 0.010	0.997 ± 0.030	1.951 ± 0.100	91.80	99.70	97.55
Artificial seawater diluted twice	0	0.450 ± 0.010	0.973 ± 0.030	1.959 ± 0.090	90.00	97.30	97.95
Artificial seawater diluted four times	0	0.484 ± 0.010	0.974 ± 0.040	1.866 ± 0.110	96.80	97.40	93.30

**Table 4:** The allowable coexisting ions in 0.5 mg/L ammonium water (measurement error less than 10% was considered as no interference).

Ions	Coexisting Content mg/L	Ions	Coexisting Content mg/L	Ions	Coexisting Content mg/L
Ca^2+^	1400	Cd^2+^	250	As^3+^	25
Mg^2+^	1800	Cr^6+^	25	SO4^2^^−^	10,000
Al^3+^	250	Mo^6+^	1250	PO4^3−^	1250
Mn^2+^	250	Co^2+^	6.25	NO_3_^−^	1250
Cu^2+^	31.25	Ni^2+^	250	NO2^−^	250
Pb^2+^	250	V^5+^	250	F^−^	1250
Zn^2+^	250	Ag^+^	6.25	Cl^−^	23,778

**Table 5:** Comparison of national method and ammonium ion test kit method.

Item	Sample	National Method	Kit Method	t Test	
				T	*ρ*
Protein content (g/100 g)	Milk powder	18.50 ± 0.50	18.57 ± 0.64	0.24	0.82
	Rice flour	6.02 ± 0.19	5.96 ± 0.28	0.49	0.64
	Wheat flour	10.38 ± 0.22	10.36 ± 0.88	0.49	0.96
	Soy	32.05 ± 0.75	32.40 ± 0.97	0.70	0.50
	Banana	3.27 ± 0.14	3.32 ± 0.19	0.56	0.59
	Milk	3.47 ± 0.25	3.62 ± 0.25	1.09	0.30
	Fish food	9.90 ± 0.28	10.19 ± 0.20	2.09	0.06
	Chicken food	10.82 ± 0.07	10.87 ± 0.06	1.40	0.19
	Dog food	20.85 ± 3.06	20.07 ± 1.61	0.55	0.59

## Data Availability

Not applicable.
